# Methylthioadenosine phosphorylase deficiency in tumors: A compelling therapeutic target

**DOI:** 10.3389/fcell.2023.1173356

**Published:** 2023-04-05

**Authors:** Na Fan, Yi Zhang, Suyun Zou

**Affiliations:** ^1^ Department of Stomatology, The Fourth Affiliated Hospital of China Medical University, Shenyang, China; ^2^ Department of Anesthesiology, Shengjing Hospital of China Medical University, Shenyang, China; ^3^ Department of Urology, The Fourth Affiliated Hospital of China Medical University, Shenyang, China

**Keywords:** MTAP, tumor, glioma, CDKN2A, methionine salvage pathway

## Abstract

The methionine salvage pathway is responsible for recycling sulfur-containing metabolites to methionine. This salvage pathway has been found to be implicated in cell apoptosis, proliferation, differentiation and inflammatory response. Methylthioadenosine phosphorylase (MTAP) catalyzes the reversible phosphorolysis of 5′-methylthioadenosine, a by-product produced from polyamine biosynthesis. The MTAP gene is located adjacent to the cyclin-dependent kinase inhibitor 2A gene and co-deletes with CDKN2A in nearly 15% of tumors. Moreover, MTAP-deleted tumor cells exhibit greater sensitivity to methionine depletion and to the inhibitors of purine synthesis. In this review, we first summarized the molecular structure and expression of MTAP in tumors. Furthermore, we discussed PRMT5 and MAT2A as a potential vulnerability for MTAP-deleted tumors. The complex and dynamic role of MTAP in diverse malignancies has also been discussed. Finally, we demonstrated the implications for the treatment of MTAP-deleted tumors.

## Introduction

The methionine salvage pathway is a universal pathway recycling sulfur-containing metabolites to methionine (Avila et al., 2004). The reversible phosphorolysis of 5′-methylthioadenosine (MTA), a by-product produced from polyamine biosynthesis, is catalyzed by methylthioadenosine phosphorylase (MTAP) (Albers, 2009). Therefore, this pathway is also named as the MTA cycle. MTA can be converted to methionine, which can be used as a source of methionine, sulfur, or purine (Sauter et al., 2013). This salvage reaction widely involves in cell apoptosis, survival, differentiation and inflammatory response (Avila et al., 2004).

**FIGURE 1:**
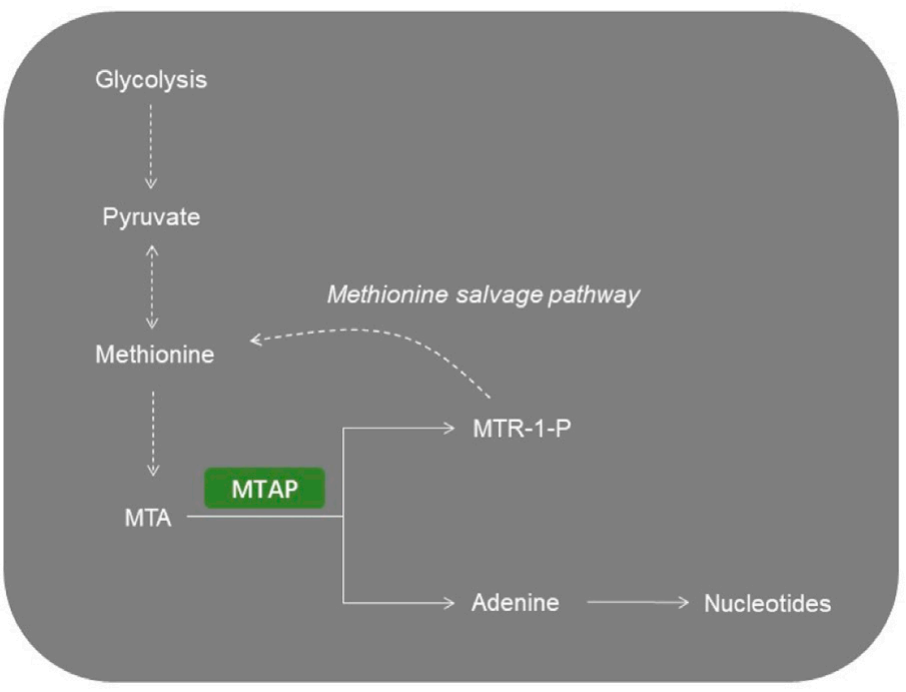
Representative image of MTAP-related metabolic pathways. MTA, 5′-methylthioadenosine; MTAP, methylthioadenosine phosphorylase; MTR-1-P, 5′-methylthioribose-1-phosphate.

The MTAP gene, located at chromosome 9p21, is close to the cyclin-dependent kinase inhibitor 2A (CDKN2A) gene and co-deletes with CDKN2A in nearly 15% of tumors (Carrera et al., 1984; Mavrakis et al., 2016). MTAP catalyzed the conversion of MTA to adenine and 5′-methylthioribose-1-phosphate, and further recycling into adenine and methionine. MTAP is abundant in normal cells but is deficient in many cancers (Kamatani and Carson, 1980; Nobori et al., 1993). In normal cells, MTAP catalyzes the conversion of MTA to adenine and 5-methylthioribose 1-phosphate. Next, adenine is converted to AMP and 5-methylthioribo-1-phosphate is to methionine. However, adenine and methionine cannot be recovered from cellular MTA in MTAP-null cells. Thus, MTAP-null cells show greater sensitivity to methionine depletion and to the inhibitors of purine synthesis. It has been shown that malignant tumor cell lines with MTAP deficiency could be targeted when purine synthesis was blocked and MTA was the only exogenous source of purines (Kamatani et al., 1981). Because of its importance in coupling the purine salvage pathway to polyamine synthesis, making MTAP a potential therapeutic target (Figure 1).

## Molecular structure and expression of MTAP

MTAP activity was initially identified from rat ventral prostate (Pegg and Williams-Ashman, 1969). It has been first discovered that certain mouse malignant hematopoietic cell lines lacked MTAP activity. MTAP is a trimeric enzyme made up of three identical subunits, sharing similarity to members of the mammalian purine nucleoside phosphorylase (PNP) family of trimeric enzymes. PNP catalyzes the phosphorolysis of the 6-oxopurine nucleosides, while MTAP targets 6-aminopurine nucleoside substrates. MTAP accepts 5′-deoxyadenosine and 6-aminopurine nucleosides containing a halogen, haloalkyl or alkylthio group at the 5′position of the sugar as substrates. The active site of MTAP contains three distinct regions including the base-, methylthioribose- and sulfate/phosphate-binding sites (Appleby et al., 1999).

MTAP loss has been commonly observed in multiple malignancies, including gliomas, pancreatic cancer, mesothelioma and non-small cell lung cancer (NSCLC). The loss of enzyme function in these cells is now believed to be a result of homozygous deletions on chromosome 9 of the closely linked MTAP and p16/MTS1 tumor suppressor genes. Thus, when MTAP is deficient, cells are failed to recycle adenine back into nucleotides. Ultimately, the rate of AMP synthesis is elevated. Herein, the MTAP-deleted cells are more susceptible to chemotherapy that selectively target the purine synthesis (Table 1).

**TABLE 1:** Current therapeutic strategies for treating MTAP-deficient tumors.

Strategy	Drugs	Tumor	Effect	Synergistic combinations	References
PRMT5 inhibitor	EPZ015666	NSCLC	Anti-proliferation		Kryukov et al. (2016)
		Melanoma			
		Breast			
	EPZ015666	Pancreatic cancer	Anti-proliferation		Driehuis et al. (2019)
	PF-06939999	NSCLC	Anti-proliferation		Jensen-Pergakes et al. (2022)
Type I PRMT inhibitor	GSK3368715	Solid and hematological malignancies	Anti-proliferation	PRMT5 inhibitor (GSK3203591)	Fedoriw et al. (2019)
	MS023	Lung cancer	Anti-proliferation	PRMT5 inhibitor (EPZ015666)	Gao et al. (2019)
		Pancreatic cancer			
MAT2A	AGI-24512	Esophageal cancer	Anti-proliferation	Taxane	Kalev et al. (2021)
		Lung cancer			
		Pancreatic cancer			
	AG-270	Esophageal cancer	Anti-proliferation	Taxane	Kalev et al. (2021)
		Lung cancer			
		Pancreatic cancer			

## PRMT5 and MAT2A as a potential vulnerability for MTAP-deleted tumors

Protein arginine methyltransferases (PRMTs) participates in the catalyzation of arginine methylation, and PRMT5 is an important member of PRMT family (Lee et al., 2021). PRMT5 play critical roles in various biological processes and regulatory pathways in transcription, translation, cell cycle and metabolic adaptation by regulating histone methylation of related proteins (Zhu and Rui, 2019). It has been demonstrated that the growth of MTAP-lacked tumor cells is abrogated by PRMT5 inhibition, making PRMT5 a synthetic lethal target for MTAP-lacked tumors (Mavrakis et al., 2016). Besides, another study focusing on the genomic profiling and functional characterization of tumor cell lines revealed that MTAP loss relied on PRMT5. Another genomic screening indicated the top genes that are synthetic lethal with MTAP loss, namely, methionine adenosyltransferase 2α (MAT2A), PRMT5 and RIOK (Marjon et al., 2016). These findings assist to identify synthetic lethal target of PRMT5 in MTAP-deleted tumors.

Upon MTAP deletion, MTA accumulation led to synthetic lethality of PRMT5. This metabolite accumulation in MTAP-deficient tumor cells led to sensitivity to PRMT5 targeting (Marjon et al., 2016). MTAP loss in MTAP-expressing tumor cells results in increased sensitivity to PRMT5 inhibition, while MTAP re-expression in MTAP-deleted tumor cells could rescue PRMT5 reliance. In MTAP-deficient tumors, MTA accumulation led to a hypomorphic state of PRMT5 that is selectively sensitized to PRMT5 deletion (Mavrakis et al., 2016). MTA could selectively target enzymatic activity of PRMT5. Both MTA uptake or PRMT5 inhibitor resulted in decreased cell viability in MTAP-deleted tumor cells compared with MTAP-expressing tumor cells (Kryukov et al., 2016).

**FIGURE 2:**
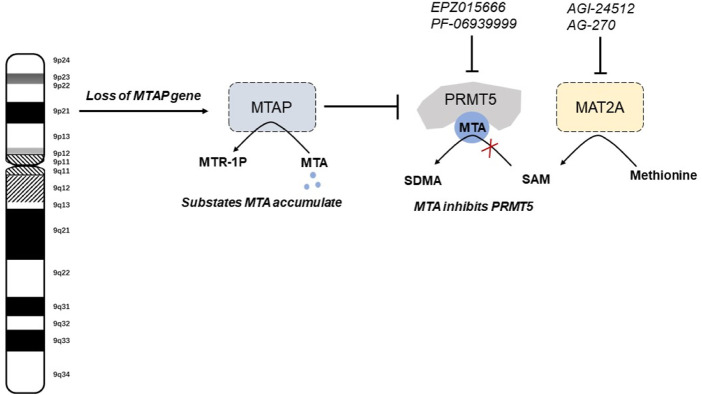
The mechanisms of synthetic lethal vulnerabilities in MTAP-deleted tumors. MTA, 5′-methylthioadenosine; MTR-1-P, 5′-methylthioribose-1-phosphate. SAM, S-adenosylmethionine; sDMA, symmetric dimethylarginine.

Collateral vulnerability also extends to the upstream metabolic enzyme MAT2A. Numerous studies have pointed out that MAT2A inhibition, exhibiting a selective antiproliferative effect in tumors with MTAP deletion, is mechanistically related to PRMT5 (Kalev et al., 2021). MAT2A has emerged as a main source of methyl donor S-adenosylmethionine, and therefore serving as a crucial regulator in metabolism and epigenetics. MAT2A depletion led to impaired tumor growth and PRMT5 activity in MTAP-deleted cells (Marjon et al., 2016) (Figure 2).

## MTAP in different malignancies

**FIGURE 3:**
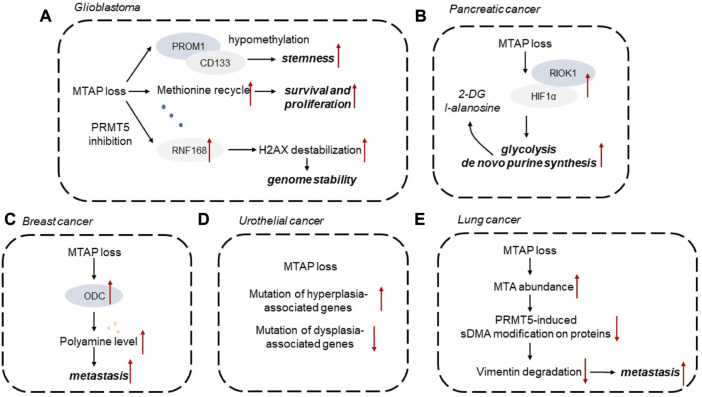
Diagrammatic representations of MTAP loss implicated in different types of cancer. **(A)** Glioblastoma, **(B)** Pancreatic cancer, **(C)** Breast cancer, **(D)** Urothelial cancer, **(E)** Lung cancer. 2-DG, 2-deoxy-d-glucose; HIF1α, hypoxia-inducible factor 1α; MTA, 5′-methylthioadenosine; ODC, ornithine decarboxylase; sDMA, symmetric dimeth.

In this section, we summarized the role of MTAP loss implicated in different types of cancer (Figure 3).

## MTAP and glioblastoma

Homozygous deletion of MTAP is often observed in glioblastoma (GBM), which represents a potential targetable vulnerability (Menezes et al., 2020). Homozygous MTAP-null cell lines showed elevated MTAP substrate metabolite, MTA. MTA accumulation suppresses PRMT5 activity, making MTA-depleted cells more responsive to PRMT5 and MAT2A inhibition. *In vivo* studies have confirmed that MTA produced from MTAP-deficient GBM cells does not significantly accumulate MTA, because MTAP-expressing stroma secretes MTA (Barekatain et al., 2021). Collectively, the metabolic differences between *in vitro* models and *in vivo* tumors should be explored for the development of precise therapeutic strategies for the treatment of GBM with MTA deficiency.

Aberrant DNA methylation has been identified as one of the emerging hallmarks of tumor cells. It has been established that MTAP deficiency could shape the DNA methylome landscape. For instance, MTAP-deleted astrocytes with high PROM1 expression display downregulated levels of DNA methylation in the PROM1 promoter. Some mutated metabolic enzymes, like IDH1/2, FH, and SDH, could elevate the levels of aberrant metabolites, which further act on epigenetic regulators to alter the DNA methylome status. However, the effects of MTAP loss on DNA methylation are still unknown and remain to be explored. Recent study also discovered that MTAP deficiency in GBM is correlated with tumor stemness of GBM cells. MTAP deficiency in GBM is correlated with altered expression of CD133 and stemness markers. MTAP loss leads to hypomethylation of PROM1/CD133-related stemness networks to mediate glioma stem-like cell formation, providing a promising therapeutic opportunity for GBM (Hansen et al., 2019). MTAP deficiency, as a genetic alteration in GBM, mediates tumor cell response to DNA damage. In MTAP-deleted GBM cells, PRMT5 inhibition existed and impaired RNF168 expression, leading to destabilization of H2AX by E3 ubiquitin ligase SMURF2. This PRMT5-RNF168-SMURF2 axis in GBM cells had significant effect on the proteostasis of H2AX, leading to altered aberrant H2AX levels for maintaining genome stability (Du et al., 2019). Methionine is required for cell survival and proliferation of GBM. MTAP loss changed methionine metabolism in GBM and led to increased consumption of methionine, considering that MTAP loss endowing GBM cells impaired ability to recycle methionine (Palanichamy et al., 2016). In the cohort of patients with GBM, MTAP loss is associated with enhanced infiltration of M2 macrophages. It has been demonstrated that MTA, as a product of MTAP loss, could activate the adenosine A2B receptor to increase the infiltration of M2-like macrophages to exert immunosuppressive effect (Hansen et al., 2022).

## MTAP and pancreatic cancer

MTAP loss has been observed in approximately 20% of pancreatic tumors. MTAP loss has been commonly observed in pancreatic tumors, and is correlated with elevated activity of ornithine decarboxylase (ODC), the rate-limiting enzyme in polyamine synthesis (Subhi et al., 2004). The ability of MTAP to suppress pancreatic tumor growth depends on its effect on polyamine production. MTAP loss also mediates metabolic adaptation of pancreatic tumor cells to increased glycolytic phenotype and *de novo* purine synthesis. Mechanistically, MTAP deletion enhances hypoxia-inducible factor 1α (HIF1α) expression depending on post-translational modification. RIO kinase 1 (RIOK1), a kinase upregulated in MTAP-deleted pancreatic tumor cells, could interact with and phosphorylate HIF1α to affect its stability. Glycolysis inhibitor 2-deoxy-d-glucose (2-DG) and purine synthesis inhibitor l-alanosine could synergistically exert anti-tumor effect on MTAP-deficient pancreatic cancer cells (Hu et al., 2021). Pancreatic cancer organoids were also used to verify that PRMT5 inhibition effectively targets MTAP-deficient tumors. PRMT5 inhibitor EZP015556 can be effective in both MTAP-deficient and a subset of MTAP-proficient organoids, both exhibiting high MTA levels. And the MTAP status of pancreatic tumors is correlated to patient response (Driehuis et al., 2019).

## MTAP and breast cancer

Breast cancer has been categorized to different subtypes according to the expression levels of molecular markers. Compared with triple-negative breast cancer, MTAP expression level is upregulated in Luminal A subtype, indicating MTAP deficiency observed in more malignant tumor subtypes (de Oliveira et al., 2016). In patients with breast cancer, MTAP deficiency is associated with poor survival outcomes. It has been demonstrated that MTAP expression could abrogate a series of malignant behaviors of breast cancer cells, including tumor angiogenesis, tumor growth and metastasis. Mechanistically, loss of MTAP activates ODC and elevate polyamine levels. In MTAP-deficient breast tumor cells, selective ODC inhibitor difluoromethylornithine (DFMO) could exert metastasis-promoting effect. Conversely, additional putrescine could impair the aggressive phenotypes of MTAP-overexpressing breast tumor cells. Herein, MTAP status is tightly related to the metastatic potential of BC cells by affecting ODC activity, which may provide promising therapeutic strategies for breast cancer (Zhang et al., 2022).

## MTAP and urothelial cancer

Molecular analysis of urothelial cancer cell lines illustrated that MTAP loss has been observed in 36% of bladder cancer cell lines (Nickerson et al., 2017). MTAP-deleted urothelial tumors exhibit more frequent mutation in hyperplasia-associated genes, such as FGFR3 and PI3KCA, whereas less common of mutations of dysplasia-associated genes (Alhalabi et al., 2021). A single arm clinical trial has evaluated the overall response rate of pemetrexed in MTAP-deficient urothelial cancer patients. Compared with patients with MTAP-proficient tumors, patients with MTAP-deficient tumors are more likely to respond to pemetrexed. Thus, a collateral lethal combination of MTAP deficiency and purine abrogation may provide potential therapeutic strategy for urothelial cancer (Alhalabi et al., 2022). When compared with MTAP-proficient urothelial tumor, MTAP-deleted urothelial tumors exhibit increased amount of targetable genetic molecules and reduced predictive biomarkers for immunotherapy (Basin et al., 2022).

## MTAP and lung cancer

MTAP has been identified as an independent prognosis marker for patients with lung tumors. MTAP deficiency is correlated with shorter survival outcomes, and the homologous loss of MTAP and p16 expression is associated with poor survival in NSCLC patients (Su et al., 2014). In MTAP-deleted lung tumor cells, MTA abundance could abrogate PRMT5-induced symmetric dimethylarginine (sDMA) modification on proteins. MTAP has been found to exert its anti-tumor effects on tumor initiation and metastasis. MTAP deletion leads to reduced level of demethylation mediated by vimentin. In MTAP-deficient cells, lower sDMA modification on protein reduces the degradation of vimentin, therefore increasing vimentin expression and ultimately enhancing tumor metastasis (Chang et al., 2022).

## Implications for the treatment of MTAP-deleted tumors

MTAP loss is a frequently observed in a series of tumors and a potential molecular target for therapy. Collectively, more efforts have been paid to explore novel anti-tumor strategies for MTAP-deficient tumors, leading to the identification of multiple synthetic lethal targets, including MAT2A and PRMT. The PRMT5-MTA complex has emerged as a novel synthetical lethal target for MTAP-deficient tumors. MRTX1719 is a potent and selective inhibitor of the PRMT5-MTA complex and specifically impairs PRMT5 activity in MTAP-deficient cells compared to MTAP-proficient cells (Smith et al., 2022). Upon MAT2A abrogation, DNA damage and mitotic defects occur in HCT116 MTAP-deficient cells, indicating the combination of AG-270 (MAT2A inhibitor) and antimitotic taxanes as an option for cancer treatment (Kalev et al., 2021). AG-270 has been currently tested in a phase I trial in patients with MTAP-deficient solid tumors and lymphomas.

Another potential therapeutic strategy for MTAP-deficient tumors is to combine toxic purine analogs such as 6′-thioguanine or 2′-fluoroadenine (2FA) with MTA. Mechanistically, MTAP mediates the conversion of MTA to adenine and inhibit purine analogs to nucleotides, to prevent the toxicity of purine in MTAP-proficient cells, whereas MTAP-deficient tumor cells lack the protection. The 2FA + MTA combination inhibits tumor growth in multiple MTAP-deficient tumor models, suggesting 2FA + MTA as a promising combination for treating MTAP-deleted tumors (Tang et al., 2018).

Besides, MTAP loss has been demonstrated to be essential for tumor immunotherapy. It has been found that 9p21 loss endows tumors with tumor immune desertification, illustrated as lower levels of tumor-infiltrating leukocytes and impaired activation of immune cell. Moreover, patients with 9p21 loss displays decreased response rates to immunotherapy. 9p21 loss could synergize with PD-L1 positivity for patient stratification (Han et al., 2021). It has also been demonstrated that TAP loss could reprogram tumor immune microenvironment towards a tumor-favorable phenotype. MTAP loss is accompanied with elevated expression of PD-L1 (Chang et al., 2023). Collectively, MTAP loss reprogram the tumor immune microenvironment, providing advantages for tumor development and immune evasion.

Type I protein PRMTs catalyze asymmetric dimethylation of arginines on proteins. A new combination regimen of type I and type II PRMT inhibitors as anti-tumor strategy has been proposed. GSK3368715 has been identified as a potent type I PRMT inhibitor, exhibiting anti-tumor effects in multiple tumor models. PRMT5 deletion could exert synergistic effect on tumor proliferation in the combined treatment with GSK3368715. Besides, MTAP deficiency led to accumulation of 2-methylthioadenosine, an inhibitor of PRMT5, which was associated with sensitivity to GSK3368715 in tumor cells (Fedoriw et al., 2019). In addition, combination of PARP inhibitors and type I PRMT inhibitors has also been proposed as a novel therapeutic strategy for MTAP-deleted NSCLC (Dominici et al., 2021).

## Conclusion

Collectively, MTAP loss may be a promising therapeutic target for developing novel strategy of cancer therapy. MTAP-deficient cells display greater sensitivity to methionine depletion and to the inhibitors of purine synthesis. Thus, MTAP-deficient cells could be selectively killed when *de novo* purine synthesis was inhibited and MTA was the only exogenous source of purines. Moreover, PRMT5 and MAT2A has been identified as a potential vulnerability for MTAP-deficient tumors based on the results from several large-scale screening and pharmacological study. MTAP loss has been found to be correlated with the prognosis of multiple malignant tumors. Herein, MTAP loss may provide promising therapeutic target and prognostic biomarker for tumors, which still needs further investigations.
